# Supplementary figures and images for: Vegetation–soil–microbiota dynamics across a 50-year reconstructed grassland chronosequence on the Loess Plateau of China
Source: PeerJ. 2024 Dec 20;12:e18723. doi: 10.7717/peerj.18723 (PMC11665427; doi:10.7717/peerj.18723)

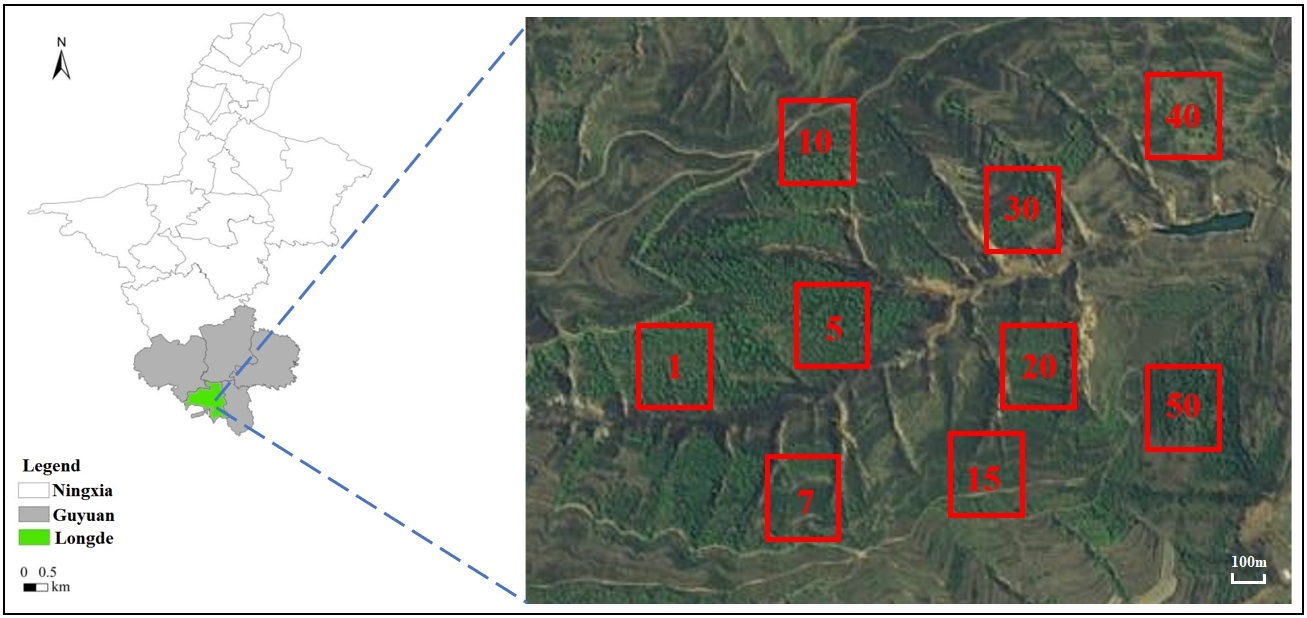

Supplement: Supplemental Information 1 [file peerj-12-18723-s001.jpg]

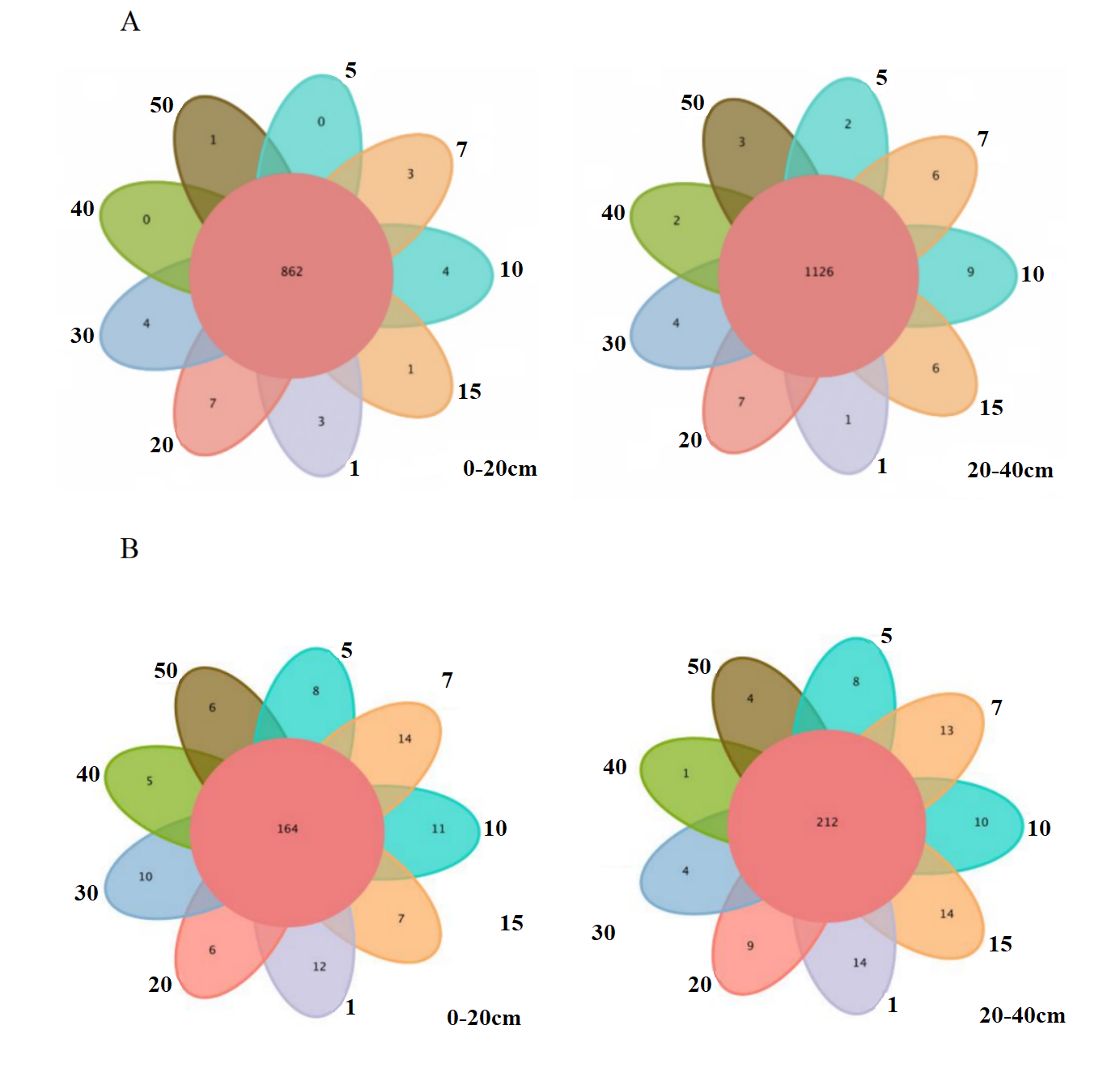

Supplement: Supplemental Information 2 — Different colors represents different stand ages. [file peerj-12-18723-s002.png]

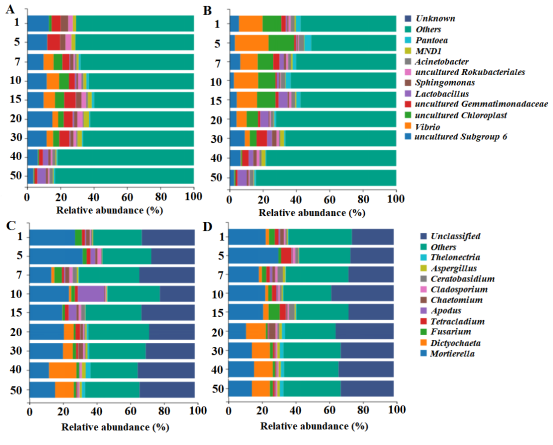

Supplement: Supplemental Information 3 [file peerj-12-18723-s003.png]

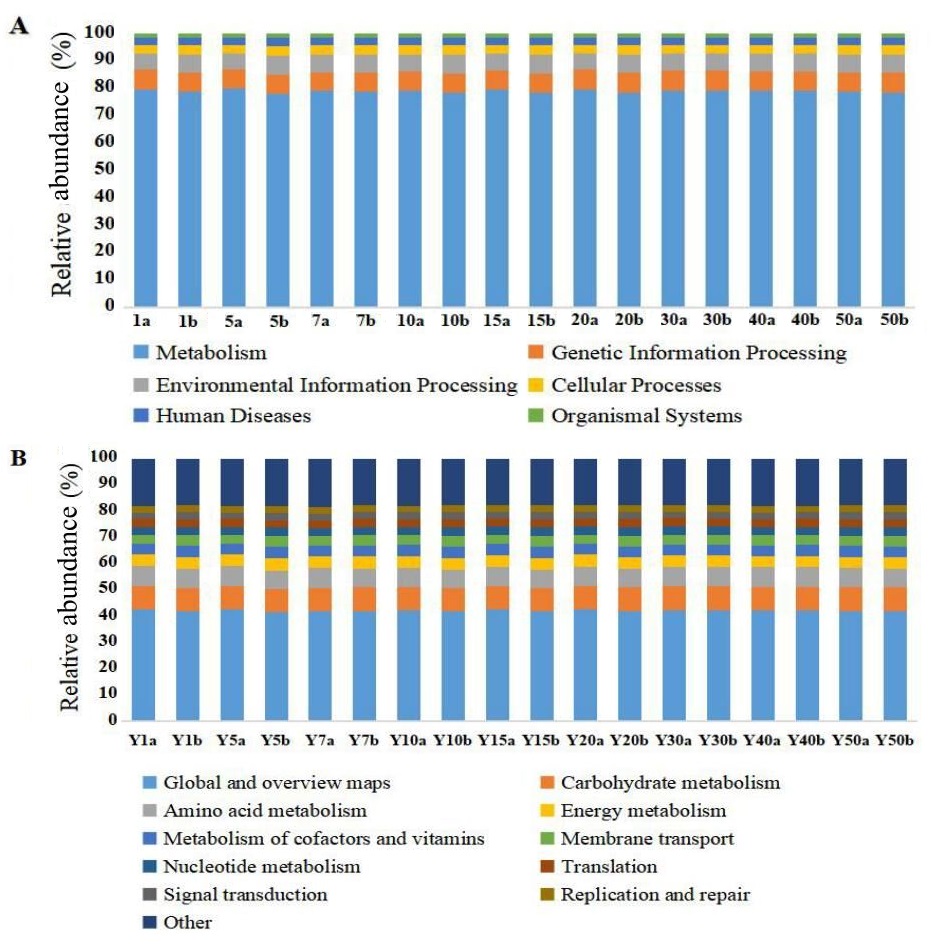

Supplement: Supplemental Information 4 — a: 0–20 cm soil depth; b: 20–40 cm soil depth. [file peerj-12-18723-s004.jpg]
