# Supplementary material for: Vegetation–soil–microbiota dynamics across a 50-year reconstructed grassland chronosequence on the Loess Plateau of China
Source: PeerJ. 2024 Dec 20;12:e18723. doi: 10.7717/peerj.18723 (PMC11665427; doi:10.7717/peerj.18723)
Supplement: Supplemental Information 5 — Different letters in the same row indicate significant differences among the age groups (P < 0.05). [file peerj-12-18723-s005.docx]

**TABLE S1** Alfalfa production performance in reconstructed grassland stands with different ages (Mean ± standard deviation).

| Stand age  (year) | Alfalfa production performance | | | | | |
| --- | --- | --- | --- | --- | --- | --- |
|  | Height (cm) | Stem diameter (cm) | Fresh weight (kg/hm^2^) | Dry weight (kg/hm^2^) | Dry-to-fresh ratio (%) | Stem-to-leaf ratio (%) |
| 1 | 25.50±3.13d | 1.97±0.15e | 10107.92±322.07bc | 2901.28±112.11bc | 28.30±4.55a | 0.82±0.09b |
| 5 | 90.60±7.22b | 3.02±0.18ab | 8110.12±277.89bc | 2910.30±190.50bc | 37.52±3.07a | 1.06±0.11b |
| 7 | 104.00±6.68a | 3.62±0.23a | 16810.18±586.17a | 5340.83±90.80a | 32.05±4.56a | 2.61±0.24a |
| 10 | 30.15±5.57d | 2.50±0.31d | 1808.22±622.09cd | 502.33±37.66cd | 30.44±5.06a | 0.80±0.08b |
| 15 | 76.12±10.56c | 3.00±1.00bc | 10099.71±278.30bc | 4021.05±99.75ab | 44.80±2.30a | 1.10±0.22b |
| 20 | 80.30±12.32c | 3.40±1.12abc | 2507.53±823.16bcd | 1100.88±107.82cd | 47.32±5.51a | 1.62±0.48b |
| 30 | 70.45±6.24c | 2.98±0.79ab | 5514.27±123.74bcd | 506.56±70.50d | 25.80±2.88a | 1.10±0.29b |
| 40 | 88.00±5.54b | 3.00±0.87bc | 540.33±76.97d | 180.47±34.65d | 32.16±1.45a | 1.22±0.44b |

Different letters in the same row indicate significant differences among the age groups (*P* < 0.05).
