# Supplementary material for: Vegetation–soil–microbiota dynamics across a 50-year reconstructed grassland chronosequence on the Loess Plateau of China
Source: PeerJ. 2024 Dec 20;12:e18723. doi: 10.7717/peerj.18723 (PMC11665427; doi:10.7717/peerj.18723)
Supplement: Supplemental Information 6 — Values followed by different letters are significantly different among the age groups or between the soil depths at P < 0.05. [file peerj-12-18723-s006.docx]

**TABLE S2** Changes in bacterial alpha-diversity in the rhizosphere of alfalfa with different stand ages.

| Stand age (year) | Soil depth (cm) | ACE | Chao1 | Simpson | Shannon |
| --- | --- | --- | --- | --- | --- |
| 1 | 0–20 | 1530.87a | 1578.60a | 0.98ab | 9.06a |
|  | 20–40 | 1475.64ab | 1418.18ab | 0.95c | 7.34b |
| 5 | 0–20 | 1523.92a | 1566.72a | 0.99a | 9.03a |
|  | 20–40 | 1454.49ab | 1458.91ab | 0.94c | 6.75b |
| 7 | 0–20 | 1494.51a | 1520.96a | 0.95bc | 8.13a |
|  | 20–40 | 1629.16a | 1457.51ab | 0.97abc | 7.68ab |
| 10 | 0–20 | 1523.74a | 1530.71a | 0.99abc | 8.19a |
|  | 20–40 | 1421.00ab | 1331.77ab | 0.95bc | 6.85b |
| 15 | 0–20 | 1516.28a | 1545.85a | 0.99abc | 8.17a |
|  | 20–40 | 1513.21ab | 1424.98ab | 0.96abc | 7.27b |
| 20 | 0–20 | 1524.52a | 1543.85a | 0.97abc | 8.61a |
|  | 20–40 | 1319.73b | 1545.91ab | 0.98ab | 7.63ab |
| 30 | 0–20 | 1560.42a | 1590.21a | 0.99a | 8.06a |
|  | 20–40 | 1608.73a | 1541.94ab | 0.99a | 8.48a |
| 40 | 0–20 | 1600.64a | 1637.60a | 0.99a | 9.07a |
|  | 20–40 | 1514.56ab | 1572.21a | 0.99a | 8.77a |
| 50 | 0–20 | 1484.87a | 1598.67a | 0.99a | 8.73a |
|  | 20–40 | 1307.73b | 1406.53ab | 0.99a | 8.50a |

Values followed by different letters are significantly different among the age groups or between the soil depths at *P <* 0.05.
