# Supplementary material for: Vegetation–soil–microbiota dynamics across a 50-year reconstructed grassland chronosequence on the Loess Plateau of China
Source: PeerJ. 2024 Dec 20;12:e18723. doi: 10.7717/peerj.18723 (PMC11665427; doi:10.7717/peerj.18723)
Supplement: Supplemental Information 7 — Values followed by different letters are significantly different among the age groups or between the soil depths at P < 0.05. [file peerj-12-18723-s007.docx]

**TABLE S3** Changes in fungal alpha-diversity in the rhizosphere of alfalfa with different stand ages.

| Stand age (year) | Soil depth (cm) | ACE | Chao1 | Simpson | Shannon |
| --- | --- | --- | --- | --- | --- |
| 1 | 0–20 | 713.39bc | 549.64b | 0.91a | 5.52a |
|  | 20–40 | 897.36bc | 629.53ab | 0.94ab | 6.25a |
| 5 | 0–20 | 799.77bc | 672.87ab | 0.95a | 5.88a |
|  | 20–40 | 875.64bc | 677.77ab | 0.97a | 5.70a |
| 7 | 0–20 | 951.41abc | 663.29ab | 0.97a | 6.47a |
|  | 20–40 | 1192.89ab | 744.35a | 0.94ab | 6.41a |
| 10 | 0–20 | 646.50c | 545.56b | 0.89a | 5.37a |
|  | 20–40 | 797.99bc | 675.61ab | 0.89b | 5.87a |
| 15 | 0–20 | 774.55bc | 646.56ab | 0.96a | 6.17a |
|  | 20–40 | 797.38bc | 689.37ab | 0.95ab | 5.88a |
| 20 | 0–20 | 775.8bc | 633.64ab | 0.93a | 5.74a |
|  | 20–40 | 943.34ab | 701.97a | 0.95ab | 5.99a |
| 30 | 0–20 | 798.62bc | 688.12ab | 0.95a | 6.31a |
|  | 20–40 | 819.62bc | 617.66ab | 0.94ab | 6.34a |
| 40 | 0–20 | 1038.92ab | 823.03ab | 0.94a | 6.32a |
|  | 20–40 | 1040.80ab | 773.19ab | 0.95ab | 6.04a |
| 50 | 0–20 | 1252a | 853.63a | 0.95a | 6.48a |
|  | 20–40 | 911.75ab | 712.25ab | 0.91b | 6.30a |

Values followed by different letters are significantly different among the age groups or between the soil depths at *P <* 0.05.
