# Supplementary material for: Vegetation–soil–microbiota dynamics across a 50-year reconstructed grassland chronosequence on the Loess Plateau of China
Source: PeerJ. 2024 Dec 20;12:e18723. doi: 10.7717/peerj.18723 (PMC11665427; doi:10.7717/peerj.18723)
Supplement: Supplemental Information 8 — *P < 0.05 and ** P < 0.01. [file peerj-12-18723-s008.docx]

**TABLE S4** Pearson correlation coefficients between rhizosphere microbial diversity indices and soil quality variables in reconstructed grasslands.

| Soil quality variable | Bacteria | | | | Fungi | | | |
| --- | --- | --- | --- | --- | --- | --- | --- | --- |
|  | ACE | Chao1 | Simpson | Shannon | ACE | Chao1 | Simpson | Shannon |
| Available P | -0.048 | 0.129 | 0.224 | 0.191 | -0.013 | 0.205 | 0.020 | 0.056 |
| Total P | 0.144 | 0.350^**^ | 0.402^**^ | 0.402^**^ | 0.162 | 0.375^**^ | 0.115 | 0.164 |
| Available K | -0.088 | 0.081 | 0.003 | 0.004 | 0.076 | 0.125 | -0.008 | 0.050 |
| Porosity | 0.170 | 0.232 | 0.121 | 0.155 | -0.032 | 0.089 | -0.163 | -0.046 |
| Field capacity | -0.053 | -0.121 | -0.151 | -0.118 | 0.139 | -0.011 | 0.107 | 0.018 |
| Bulk density | 0.227 | 0.217 | 0.262 | 0.266 | 0.355^**^ | 0.242 | 0.137 | 0.183 |
| Total N | 0.195 | 0.234 | 0.216 | 0.177 | -0.041 | 0.086 | 0.012 | 0.019 |
| Water content | 0.091 | 0.037 | -0.039 | -0.037 | -0.284^*^ | -0.276^*^ | -0.349^**^ | -0.251 |
| Available N | 0.104 | 0.291^*^ | 0.117 | 0.173 | 0.105 | 0.303^*^ | 0.140 | 0.191 |
| Total organic C | 0.097 | 0.302^*^ | 0.237 | 0.254 | -0.022 | 0.190 | -0.085 | -0.076 |
| pH | -0.048 | 0.129 | 0.224 | 0.191 | -0.013 | 0.205 | 0.020 | 0.056 |
| Total K | -0.051 | 0.192 | 0.252 | 0.255 | 0.088 | 0.198 | -0.049 | 0.072 |
| Urease | 0.186 | 0.187 | -0.053 | -0.015 | 0.103 | 0.191 | 0.198 | 0.198 |
| Sucrase | 0.225 | 0.139 | 0.152 | 0.091 | 0.032 | 0.210 | 0.149 | 0.169 |
| Phosphatase | 0.137 | 0.075 | 0.162 | 0.149 | 0.150 | 0.200 | -0.031 | 0.175 |
| Catalase | 0.099 | -0.022 | 0.188 | 0.094 | -0.125 | 0.046 | -0.072 | -0.003 |

**P <* 0.05 and ** *P <* 0.01.
